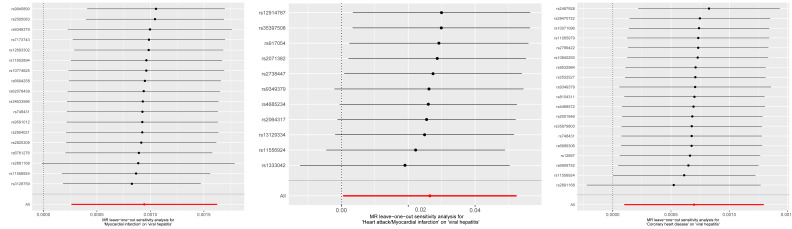# Association between coronary artery disease and viral hepatitis: Perspectives based on the 2017–2018 National Health and Nutrition Examination Survey and Mendelian randomization analyses

**DOI:** 10.1097/MD.0000000000049202

**Published:** 2026-06-05

**Authors:** Zihan Zhou, Zhuo Bai, Chang Zheng, Jing Wu, Lan Li, Aibin Cheng, Xiuli Men

**Affiliations:** a School of Basic Medical Sciences, North China University of Science and Technology, Tangshan, Hebei, China; b North China University of Technology and Healthy Affiliated Hospital, Tangshan, Hebei, China.

**Keywords:** causality, coronary artery disease, Mendelian randomization analysis, National Health and Nutrition Examination Survey, viral hepatitis

## Abstract

Coronary artery disease (CAD) is one of the leading forms of cardiovascular disease in Europe and the primary cause of death in the region, while millions of individuals in Europe remain chronically infected with viral hepatitis, resulting in a persistent disease burden. A European study has previously indicated that the prevalence of hepatitis C virus infection among hospitalized patients with ischemic heart disease is significantly higher than the prevalence observed in the general population screened locally. Given this, further clarification of the association between CAD and viral hepatitis in European populations, and its potential underlying mechanisms, holds substantial practical significance, which may guide the optimization of clinical screening and the development of targeted prevention strategies. This study aims to explore this association using data from European populations. We conducted a cross-sectional study using data from National Health and Nutrition Examination Survey 2017 to 2018 and assessed the association between coronary heart disease (CHD), angina/angina pectoris, heart attack/myocardial infarction (MI) and viral hepatitis and other hepatic disorders by multivariable weighted-adjusted logistic regression analyses. Secondly, we applied Mendelian randomization based on inverse variance weighting method and complementary methods to analyze the relationship between CHD, heart attack/MI, MI and viral hepatitis while sensitivity analyses were used to assess the pleiotropy of the results. We found that CAD may be positively associated with viral hepatitis in European populations. A total of 5501 participants were enrolled in our cross-sectional study. Logistic regression analysis showed that both CHD (odds ratio [OR; 95% confidence interval [CI]]: 2.06 [1.10–3.85]; *P* = .041) and angina/angina pectoris (OR [95% CI]: 3.81 [1.23–11.81]; *P* = .036) were significantly positively associated with viral hepatitis and other hepatic disorders. The results of bidirectional Mendelian randomization analyses supported a significant positive effect between MI (OR = 1.00094, 95% CI: 1.00026–1.00163, *P* = .007), heart attack/MI (OR = 1.027, 95% CI: 1.00065–1.054, *P* = .044), CHD (OR = 1.00070, 95% CI: 1.00010–1.0013, *P* = .022) and viral hepatitis, the sensitivity analyses further confirmed the robustness of the results.

## 1. Introduction

Viral hepatitis, as a significant global concern for public health, impacts millions of individuals worldwide due to its severe morbidity and mortality.^[1]^ Globally, about 2 million people die annually due to liver disease, of which about 1 million die from viral hepatitis and hepatocellular carcinoma.^[2]^ The burden due to viral hepatitis is very heavy. Among the 4 major global infectious diseases, the mortality rates of hepatitis B virus (HBV) and hepatitis C virus (HCV) infections are comparable to those of AIDS, tuberculosis, and malaria.^[3]^ It should be emphasized that HBV and HCV are closely linked to the development of liver cancer.^[4]^ The HBV infects nearly one-third of the world’s population, and among these individuals, of whom about 5% are persistent carriers, within this carrier group, 15 to 40% may eventually suffer from cirrhosis, cancer, or liver failure. And the majority of people with viral hepatitis do not have any visible signs or symptoms, which means that they may not be aware of the infection in their bodies, making targeted increases in screening and preventive measures all the more important.^[5]^

Coronary artery disease (CAD), also known as coronary heart disease (CHD), is a heart disease in which the blood and oxygen supply to the myocardium is insufficient, leading to myocardial dysfunction or organic lesions. CAD includes various types such as occult CAD, angina pectoris, ischemic cardiomyopathy, and myocardial infarction (MI) which is one of the most serious forms of CAD.^[6]^ CAD has been identified as a severe and potentially life-threatening complication of HCV infection.^[7]^ According to a meta-analysis by K. K. Lee et al, individuals diagnosed with HCV presented a greater susceptibility to cardiovascular ailments when compared to those without HCV infection (relative risk = 1.28, 95% confidence interval [CI]: 1.18–1.39); the risk ratio for MI was 1.13 (95% CI: 1.00–1.28) when stratified by outcome.^[8]^ Another separate study conducted by Tsai et al found that patients infected with HCV who haven’t received antiviral treatment exhibited an elevated susceptibility to acute coronary syndrome when compared to those without the infection.^[9]^ Additionally, a study by Vassalle et al revealed that HCV seropositivity independently contributed to the risk of CHD, even after accounting for other influential factors.^[10]^

Viral hepatitis could be found commonly as a comorbidity of CAD and its associated heart diseases in the above mentioned observational clinical studies, however, existing observational studies have some limitations, such as small sample size, homogeneous disease types, high influence of bias factors, under-representation, and inevitable reverse causal inference, in which it is difficult to systematically identify independent causal associations between CAD and CAD-related heart disease to viral hepatitis. The National Health and Nutrition Examination Survey (NHANES) was organized by the US Centers for Disease Control and Prevention as a cross-sectional study designed to assess the population health and nutritional status of the US population,^[11]^ which consists of participant interviews conducted in their home, clinical and laboratory examinations conducted at Mobile Examination Center, and information on demographic characteristics, health status, and body composition. The NHANES can provide data from a large, high-quality, and nationally representative sample to estimate the associations between CAD and viral hepatitis. Meanwhile, as a powerful tool in epidemiological studies, within the cross-sectional study framework, the core ideology of the Mendelian randomization (MR) approach aims to provide supportive evidence for causal relationships between specific diseases and risk factors by utilizing stable genetic markers as instrumental variables (IVs).^[12–15]^ Compared to traditional randomized controlled trials, MR methods can overcome the bias caused by confounding factors and reverse causality.^[16]^

The aim of this study was to explore the association between CAD and viral hepatitis in the general population using NHANES 2017 to 2018 data and to determine the potential causal effect of CAD on viral hepatitis using MR analysis. This will broaden the scope of screening for viral hepatitis and lay the theoretical foundation for preventive measures in the relevant population.

## 2. Materials and methods

### 2.1. Cross-sectional study in NHANES

#### 2.1.1. Research design

**Figure 1.**
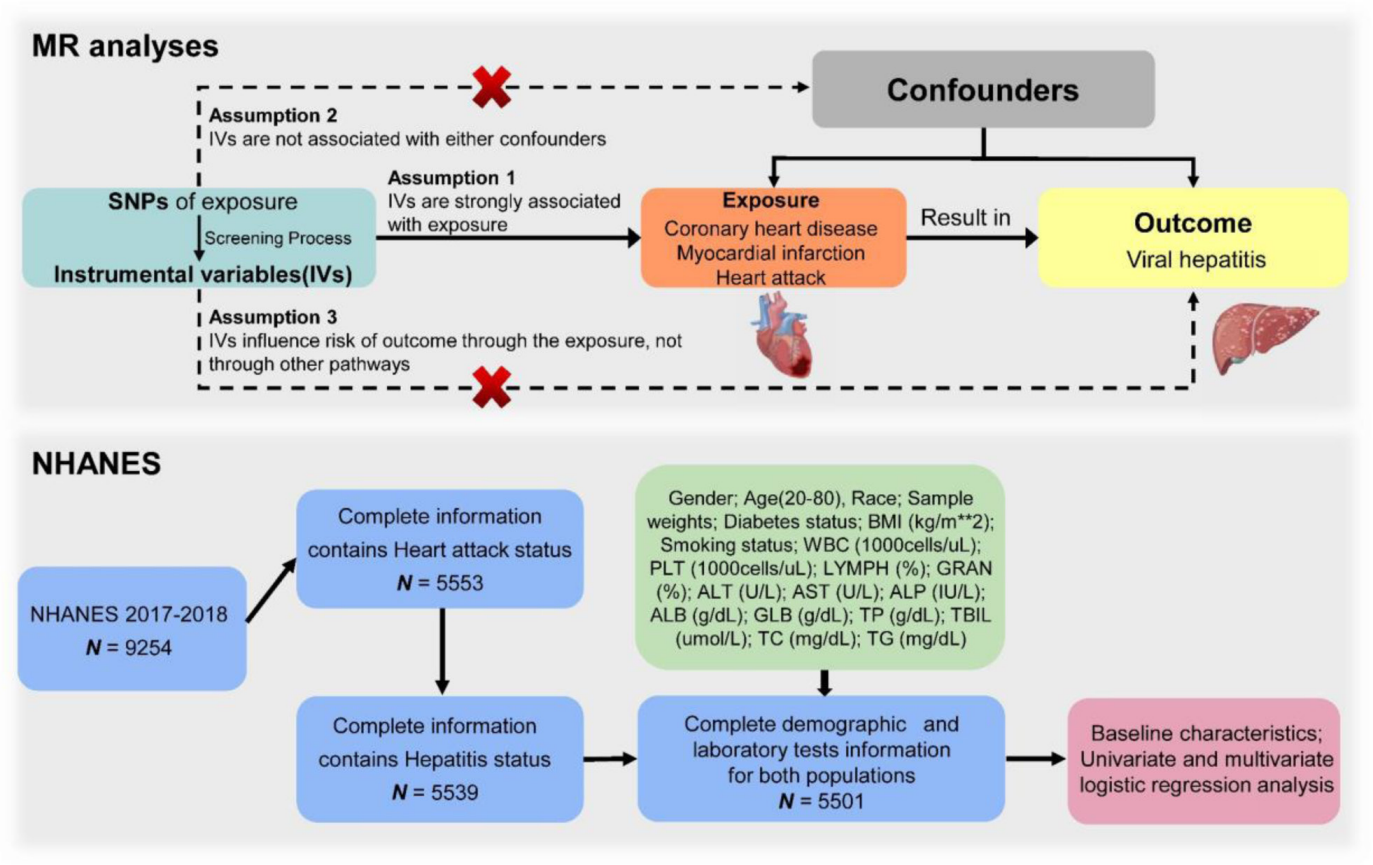
Conceptual framework of this study, which shows the main steps of the flow and the principles that should be followed. ALB = albumin, ALP = alkaline phosphatase, ALT = alanine aminotransferase, AST = aspartate aminotransferase, BMI = body mass index, GLB = globulin, GRAN = segmented neutrophils percent, IV = instrumental variable, LYMPH = lymphocyte percent, MR = Mendelian randomization, NHANES = National Health and Nutrition Examination Survey, PLT = platelet count, SNPs = single nucleotide polymorphisms, TBIL = total bilirubin, TC = total cholesterol, TG = triglycerides, TP = total protein, WBC = white blood cell count.

This study consists of 2 main parts: the cross-sectional study and the MR analysis. In the cross-sectional study, we will investigate the association between CAD and the broad category of “hepatic disorder (including hepatitis),” 9254 participants aged 20 to 80 years were selected from the 2017 to 2018 NHANES database, due to the missing data on CHD/MI and hepatic disorder status (including viral hepatitis), laboratory test data, diabetes status data, angina status data, heart disease status data, and smoking status data, after excluding 3753 participants with incomplete data, a total of 5501 participants were enrolled in this study (Fig. 1), of which 288 participants were diagnosed with hepatic disorder including viral hepatitis. We simultaneously weighted the data according to the sample weighting parameters of the NHANES database.^[17]^ The NHANES project was approved by the Ethics Review Board of the National Center for Health Statistics, all participants signed an informed consent form, and the data used in this study were obtained from its publicly available database (https:/www.cdc.gov/nchs/nhanes/index.htm), thus no additional authorization or ethical review.

#### 2.1.2. Definition of research variables in NHANES

Based on previous epidemiological studies,^[18–20]^ the factors to be included in the analysis for this research are as follows:

1. Demographic data: Age, sex, and race/ethnicity;
2. Basic physical measurements: Body mass index (BMI; kg/m^2^);
3. Laboratory tests: White blood cell count (1000 cells/µL), platelet count (1000 cells/µL), lymphocyte percent (%), segmented neutrophils percent (%), alanine aminotransferase (ALT; U/L), aspartate aminotransferase (AST; U/L), alkaline phosphatase (ALP; IU/L), albumin (g/dL), globulin (GLB; g/dL), total protein (g/dL), total bilirubin (TBIL; umol/L), total cholesterol (mg/dL), and triglycerides (mg/dL);
4. Questionnaire data: Heart attack/MI status, CHD status, angina/angina pectoris status, smoking status, and diabetes status.

Questionnaire data collection was done by interviewers using the Computer Assisted Personal Interview system at the participants’ home, whereas the collection of basic body measurements and laboratory test samples was done by professional health technicians at the Mobile Examination Centre, and the process and quality control of the laboratory tests followed the NHANES standards.^[21]^ Among them, the indicators of the laboratory test part were selected from the complete blood count, routine blood 5 classifications, and blood biochemical examination. The changes of these indicators can fully reflect the degree of liver damage, which provide an important reference basis for the diagnosis of viral hepatitis. In the questionnaire section, we defined “hepatic disorder” as the self-reported presence of any type of liver disease. According to the NHANES entry description, hepatic disorder was defined to include viral hepatitis (hepatitis A, hepatitis B, and hepatitis C); autoimmune liver disease; hereditary liver disease; drug- or drug-induced liver disease; alcoholic liver disease; nonalcoholic fatty liver disease; steatohepatitis; hepatocellular carcinoma; liver cysts; liver abscesses; hepatic fibrosis; and cirrhosis. Meanwhile, we enrolled data from a total of 3 questionnaires on CAD and its related diseases, defined as “self-reported coronary heart disease,” “self-reported angina/angina pectoris,” and “self-reported heart attack/myocardial infarction.” We also defined participants with “self-reported overlapping cardiovascular disease” as those who self-reported 2 or more of the following conditions: CHD, angina/angina pectoris, and heart attack/MI. Diabetes mellitus (DM) status was determined based on the patient’s medical history and blood glucose values, and smoking status was recorded by “self-reported history of smoking at least 100 cigarettes.” Participants who refused to answer, answered “I don’t know,” or had missing information on influencing factors were excluded from the questionnaire portion of the data.

We specifically declare that since previous surveys for hepatic disorders in the NHANES database did not cover viral hepatitis and the lack of separate descriptions of viral hepatitis samples included in the 2017 to 2018 survey, we therefore chose the entry for hepatic disorder that included the definition of viral hepatitis as the basis for grouping patients. We aim to first establish broad associations between CAD and hepatic disorder through cross-sectional studies, followed by MR analysis to provide potential evidence for causal relationships between CAD and viral hepatitis phenotypes.

#### 2.1.3. Statistical analysis of NHANES

In this study, people aged 20 and 80 years were divided into 2 groups according to the presence or absence of hepatic disorders. In this section, participants with missing respondent information or personal details were excluded. We compared the clinical baseline information between the hepatic disorder population and non-hepatic disorder controls, where normally distributed continuous data were expressed as Mean ± SD, and comparisons between the 2 groups were analyzed using the *t*-test for two independent samples; categorical data were expressed as n (%), and the chi-square test or the Fisher precision probability test was applied for comparisons. The difference was considered statistically significant at *P* < .05. The correlation between CHD, angina/angina pectoris, Heart attack/MI and liver disease was then analyzed by univariate logistic regression. Then, we conducted a multivariable logistic regression analysis. Referencing covariate adjustment methods from existing relevant studies, we adjusted for the following covariates: age, sex, and race/ethnicity (Model 1),^[22]^ BMI, smoking status, and diabetes status (Model 2),^[23]^ and laboratory test indicators related to the diagnosis of viral hepatitis and significantly different between the two groups (Model 3).^[24]^ This allowed us to investigate the impact of these covariates on the association between CAD and its related conditions with hepatic disorders. These models are presented below:

Crude model: unadjusted.

Model 1: adjusted for Sex + Age + Race/Ethnicity.

Model 2: Model 1 + BMI + Diabetes status + Smoking status.

Model 3: adjusted for ALT + ALP + AST + GLB + TBIL.

In this study, we used R 4.3.0 statistical analysis software. Based on the raw data, we used the R package “survey” to analyze the weights of the complex multistage sampling design of the NHANES database to produce unbiased and nationally representative statistics.

### 2.2. MR study

#### 2.2.1. Research design

In MR analysis, we will examine the evidence for a causal relationship between CAD and narrower category of “viral hepatitis.” Figure 1 illustrates the flowchart of our MR study. In order to accurately assess the causal effect, adherence to 3 assumptions is necessary for the MR analysis. Firstly, it is crucial that the IVs display a strong association with exposure factor. Secondly, the IVs must remain unrelated to any potential confounding factors. Lastly, the effects of the IVs on viral hepatitis should solely occur through exposure factor.^[25,26]^ Given that publicly accessible GWAS data were employed, there was no need for additional ethical approval.

#### 2.2.2. Data source

To describe CAD more comprehensively, the exposure dataset included a total of 3 CAD or CAD-related phenotypes, of which the CHD dataset was obtained from a genome-wide association meta-analysis based on the 1000 Genomes Project,^[27]^ the Heart attack/MI dataset was derived from the self-reported Non-Cancer Codes section of UKBiobank,^[28]^ and the MI dataset was integrated from 2 genome-wide association summary data.^[29]^ The outcome dataset was constructed by obtaining variables from UKBiobank through PHESANT.^[30]^

All of the above samples are from European populations to minimize bias due to race/ethnicity-related confounders, and details of each summary data are given in Table 1.

**Table 1:** Summary of genome-wide association study (GWAS) data.

Phenotype	GWAS ID	Year	Sample size	n case	n control	n snp	Population
Myocardial infarction	ebi-a-GCST011365	2021	639,221	61,505	577,716	8126,035	European
Heart attack/Myocardial infarction	ukb-a-63	2017	337,159	7735	329,424	10,894,596	European
Coronary heart disease	ieu-a-7	2015	184,305	60,801	123,504	9,455,779	European
Viral hepatitis	ukb-b-7901	2018	462,933	1024	461,909	9,851,867	European

n = number, snp = single nucleotide polymorphism.

#### 2.2.3. Selection of IVs

We used a rigorous standardized procedure to extract single nucleotide polymorphism (SNPs) that met the genome-wide significance thresholds from the GWAS summary data of the exposure factors, and to achieve a sufficiently satisfactory number of SNPs, we set the thresholds for the CHD phenotype and the Heart attack/MI phenotype set to *P* = 5 × 10^−6^ and the threshold for the MI phenotype to *P* = 5 × 10^−8^. Typically, *P* = 5 × 10^−8^ serves as the gold standard for genome-wide significance in GWAS.^[31]^ For certain emerging phenotypes or exposure factors with low heritability, strictly enforcing the *P* = 5 × 10^−8^ threshold may result in an insufficient number of included SNPs (such as fewer than 3), which significantly reducing the statistical power of MR analyses. In such cases, moderately loosening the threshold to *P* = 5 × 10^−6^ or higher is an academically accepted alternative approach.^[32–36]^

In order to ensure the absence of linkage disequilibrium aggregation between SNPs that could potentially result in biased outcomes, we identified independent SNPs by applying the “clump” method.^[37]^ These SNPs were required to have an *r*2 value <0.001 and the distance between SNPs should >10,000 kb. Additionally, we utilized the PhenoScanner (http://www.phenoscanner.medschl.cam.ac.uk) to investigate any previously reported associations between each SNP and viral hepatitis or its established risk factors (*P* < 1 × 10^−5^).^[38]^ To mitigate the influence of weak IV bias, we computed the F-statistic for each SNP using the subsequent formula: F= $R^{2}$ (N − 2)/(1 − $R^{2}$), where $R^{2}$ represents the extent of genetic variation explained by each SNP, N denotes the sample size of the exposed dataset. Any SNP with an F-statistic below 10 was deemed weak and subsequently excluded.^[39]^

After harmonizing exposure SNPs and outcome SNPs, we removed outliers with potential multiplicity through MR-pleiotropy residual sum and outlier (MR-PRESSO) method, we also excluded palindromic SNPs according to their allele frequencies.^[40]^ Meanwhile, we did not use any proxy SNPs.

#### 2.2.4. Statistical analysis for bidirectional MR

Utilizing the hypothesis that all evaluated SNPs are trustworthy IVs, the inverse variance weighted (IVW) method stands out as the utmost precise and dependable MR method. The calculation of the causal impact of each SNP involves determining the Wald ratio between the exposure effect and the corresponding outcome effect.^[41]^ Consequently, we applied a fixed-effects IVW strategy to derive comprehensive effect measures through the aggregation of causal estimations across all SNPs, The IVW method gives the most precise results when IVs are free of horizontal pleiotropy,^[42]^ and has a strong causality detection capability.^[43]^ The primary method utilized in bidirectional MR was IVW. Since the following methods were not as effective as IVW,^[44]^ the MR-Egger,^[45]^ weighted median,^[46]^ and weighted mode^[47]^ were used as auxiliary tests in the forward MR analysis. Meantime, based on the aforementioned IVs screening process, setting *P* = 5 × 10^−5^ as the threshold, viral hepatitis as the exposure factor, and 3 CAD-related phenotypes as the outcome, we performed reverse MR analysis to eliminate the interference of reverse causality to ensure the reliability of the results.

Our MR estimates for each exposure factor were expressed as odds ratio (OR) and 95% CI per log unit increase in the events of outcome. Two-sided values of *P* < .05 were considered statistically significant. We used the “TwoSampleMR” package (version 0.5.4) in R (version 4.3.0)^[39]^ to integrate and analyze the data.

#### 2.2.5. Sensitivity analysis and heterogeneity test

MR‐Egger intercept test and MR‐PRESSO global test were used to test for horizontal polytropy of the samples.^[40]^ If the MR-Egger intercept tends to zero as the sample size increases, and the MR-PRESSO method with *P*-value > .05, the selected samples are free of pleiotropy originating from genetic variation.^[48]^ In order to visually inspect pleiotropy, we also plotted the funnel plots representing the estimated effect of each SNP. Additionally, “leave-one-out” analyses, which eliminate SNPs with excessively large outliers, also applying in the sensitivity analysis step.

The heterogeneity test was conducted using Cochran *Q* test to offer proof of genetic heterogeneity resulting from directional pleiotropy or other underlying factors.^[49]^ Cochran *Q* test in MR-Egger and IVW methods has been widely used for heterogeneity testing.

The *P*-value > .05 in Cochran *Q* test indicated no heterogeneity in results.^[50]^ R Packages “Mendelian Randomization”^[43]^ and “MR-PRESSO”^[40]^ was used to complete the sensitivity analysis and heterogeneity test.

## 3. Results

### 3.1. Baseline characteristics of the study population

A total of 2664 males (48.43%) and 2837 females (51.57%) were enrolled in this study according to the established inclusion/exclusion criteria. Based on the definition of the Hepatic disorder entry, the participants were divided into 288 individuals with hepatic disorder including viral hepatitis (Hepatic disorder group) and 5213 individuals without hepatic disorder (Control group). The socio-demographic and laboratory characteristics of the study participants are summarized in Table 2, where the mean values of biochemical indicators ALT, ALP, AST, GLB, TBIL, total protein, triglycerides were significantly higher in the hepatic disorder group than in the control group (*P* < .05), and the mean value of the haematological indicator platelet count was significantly lower than that in the control group (*P* < .05), and the characteristics of these variations were in line with the results of the expected laboratory tests for viral hepatitis.

**Table 2:** Comparison of demographic and clinical characteristics of study participants.

Variables	Total (n = 5501)	Control (n = 5213)	Hepatic disorder (n = 288) (Including viral hepatitis)	Statistic	*P*
Age Mean ± SD	51.393 ± 17.785	51.024 ± 17.900	58.062 ± 13.993	t = −8.174	**<.001**
ALT Mean ± SD	22.416 ± 17.035	22.022 ± 16.438	29.545 ± 24.489	t = −5.150	**<.001**
ALB Mean ± SD	4.037 ± 0.379	4.039 ± 0.380	4.005 ± 0.353	t = 1.498	0.134
ALP Mean ± SD	79.309 ± 27.243	78.794 ± 26.938	88.628 ± 30.859	t = −5.298	**<.001**
AST Mean ± SD	22.006 ± 13.164	21.685 ± 12.713	27.812 ± 18.715	t = −5.487	**<.001**
GLB Mean ± SD	3.105 ± 0.434	3.100 ± 0.431	3.192 ± 0.474	t = −3.520	**<.001**
TBIL Mean ± SD	7.838 ± 4.630	7.780 ± 4.594	8.883 ± 5.134	t = −3.566	**<.001**
TC Mean ± SD	189.835 ± 42.008	189.750 ± 41.974	191.365 ± 42.668	t = −0.635	.526
TP Mean ± SD	7.142 ± 0.442	7.139 ± 0.442	7.197 ± 0.451	t = −2.169	**.030**
TG Mean ± SD	147.667 ± 116.302	146.371 ± 115.291	171.139 ± 131.302	t = −3.522	**<.001**
BMI Mean ± SD	29.896 ± 7.427	29.842 ± 7.434	30.867 ± 7.237	t = −2.279	**.023**
WBC Mean ± SD	7.383 ± 5.838	7.388 ± 5.971	7.295 ± 2.396	t = 0.263	.792
LYMPH Mean ± SD	31.429 ± 8.969	31.425 ± 8.963	31.517 ± 9.088	t = −0.170	.865
GRAN Mean ± SD	56.910 ± 9.626	56.928 ± 9.609	56.585 ± 9.942	t = 0.589	.556
PLT Mean ± SD	244.473 ± 66.102	245.384 ± 65.750	227.972 ± 70.291	t = 4.106	**<.001**
Sex n(%)				χ^2^ = 1.626	.202
Female	2837 (51.572)	2699 (51.774)	138 (47.917)		
Male	2664 (48.428)	2514 (48.226)	150 (52.083)		
Race/Ethnicity n(%)				χ^2^ = 45.410	**<.001**
Mexican American	723 (13.143)	666 (12.776)	57 (19.792)		
Non-Hispanic Black	1288 (23.414)	1250 (23.979)	38 (13.194)		
Non-Hispanic White	1911 (34.739)	1812 (34.759)	99 (34.375)		
Other Hispanic	512 (9.307)	463 (8.882)	49 (17.014)		
Other Race – Including Multi-Racial	1067 (19.396)	1022 (19.605)	45 (15.625)		
Diabetes n(%)				χ^2^ = 36.020	**<.001**
Prediabetes	168 (3.054)	153 (2.935)	15 (5.208)		
No	4480 (81.440)	4284 (82.179)	196 (68.056)		
Yes	853 (15.506)	776 (14.886)	77 (26.736)		
Coronary heart disease n(%)				χ^2^ = 4.521	**.033**
No	5242 (95.292)	4975 (95.434)	267 (92.708)		
Yes	259 (4.708)	238 (4.566)	21 (7.292)		
Angina/Angina pectoris n(%)				χ^2^ = 10.756	**.001**
No	5347 (97.201)	5076 (97.372)	271 (94.097)		
Yes	154 (2.799)	137 (2.628)	17 (5.903)		
Heart attack/Myocardial infarction n(%)				χ^2^ = 6.961	**.008**
No	5239 (95.237)	4974 (95.415)	265 (92.014)		
Yes	262 (4.763)	239 (4.585)	23 (7.986)		
Overlapping cardiovascular disease n(%)				χ^2^ = 5.465	**.019**
No	5311 (96.546)	5040 (96.681)	271 (94.097)		
Yes	190 (3.454)	173 (3.319)	17 (5.903)		
Smoking n(%)				χ^2^ = 11.823	**<.001**
No	3209 (58.335)	3069 (58.872)	140 (48.611)		
Yes	2292 (41.665)	2144 (41.128)	148 (51.389)		

The bolded values indicate results that meet the significance requirement of *P* < .05.

χ^2^= Chi-square test, ALB = albumin (g/dL), ALP = alkaline phosphatase (IU/L), ALT = alanine aminotransferase (U/L), AST = aspartate aminotransferase (U/L), BMI = body mass index (kg/m^2^), GLB = globulin (g/dL), GRAN = segmented neutrophils percent (%), LYMPH = lymphocyte percent (%), PLT = platelet count (1000 cells/µL), SD = standard deviation, t = t-test, TBIL = total bilirubin (umol/L), TC = total cholesterol (mg/dL), TG = triglycerides (mg/dL), TP = total protein (g/dL), WBC = white blood cell count (1000 cells/µL).

We also examined the prevalence of the 3 CAD and CAD-related diseases between the hepatic disorder group and the control group, and the results showed that the prevalence of CHD (7.292% vs 4.566%; χ^2^ = 4.521, *P* = .033), angina/angina pectoris (5.903% vs 2.628%; χ^2^ = 10.756, *P* = .001), heart attack/MI (7.986% vs 4.585%; χ^2^ = 6.961, *P* = .008) have significant differences between the groups, moreover, in the control group, 96.681% of participants had no overlapping heart disease, while 3.319% had overlapping heart disease. Within the group with hepatic disorder (including viral hepatitis), 94.097% had no overlapping heart disease, and 5.903% had overlapping heart disease. The difference in overlapping heart disease distribution between the 2 groups was statistically significant (χ^2^ = 5.465, *P* = .019). These findings suggest that there may be complex and widespread associations between CAD and viral hepatitis and other hepatic disorders.

In addition, age, bmi, race/ethnicity, diabetes, and smoking (lifestyle factors) also have significant differences between the groups (*P* < .05), while the differences of sex, albumin, total cholesterol, white blood cell count, lymphocyte percent, and segmented neutrophils percent were not statistically significant (*P* > .05).

### 3.2. Results of logistics regression analysis in NHANES

We performed univariate and multivariate logistics regression analyses for CHD, angina/angina pectoris, and heart attack/MI as risk factors for hepatic disorder respectively. Table 3 shows the correlation between the 3 CAD/CAD-related diseases and the hepatic disorder. In the crude model (univariate logistics regression analysis), we observed that both CHD (OR [95% CI]: 2.06 [1.10–3.85]; *P* = .041) and angina/angina pectoris (OR [95% CI]: 3.81 [1.23–11.81]; *P* = .036) contributed to the events of hepatic disorder significantly, although no significant association was observed between heart attack/MI and hepatic disorder (OR [95% CI]: 2.20 [1.00–4.82]; *P* = .070), but its OR may still indicate a positive effect of heart attack/MI on the events of hepatic disorder.

**Table 3:** Association between CAD/CAD-related diseases and liver condition.

Exposure	Models	n	Liver condition (Including viral hepatitis)	
			OR (95% CI)	*P*-value
Coronary heart disease	Crude model	5501	2.06 (1.10–3.85)	.041
(Sample size = 259)	Model 1	5501	1.16 (0.62–2.17)	.663
	Model 2	5501	1.10 (0.59–2.05)	.772
	Model 3	5501	2.04 (1.10–3.78)	.049
Angina/Angina pectoris	Crude model	5501	3.81 (1.23–11.81)	.036
(Sample size = 154)	Model 1	5501	2.43 (0.78–7.57)	.164
	Model 2	5501	2.32 (0.74–7.26)	.222
	Model 3	5501	3.63 (1.08–12.25)	.067
Heart attack/Myocardial infarction	Crude model	5501	2.20 (1.00–4.82)	.070
(Sample size = 262)	Model 1	5501	1.35 (0.59–3.12)	.501
	Model 2	5501	1.26 (0.53–3.02)	.629
	Model 3	5501	2.08 (0.95–4.59)	.102

CAD = coronary artery disease, CI = confidence interval, n = number, OR = odds ratio.

In multivariate logistic regression analyses, we adjusted sex, age, and race/ethnicity as covariates in model 1, on the basis of model 1, BMI, diabetes status, and smoking status were added as covariates in model 2; and several laboratory indicators related to the diagnosis of viral hepatitis were adjusted as covariates in model 3. The results showed that CHD exhibited a significant positive association with the hepatic disorder in model 3 (OR [95% CI]: 2.04 [1.10–3.78]; *P* = .049), while no significant associations were observed in the remaining models (*P* > .05), but all in the same direction (OR > 1). We noted that each risk factor showed the strongest positive effect in model 3, however, the positive effects of models 1 and 2 were attenuated, which might possibly due to the inclusion of covariates such as sex, which was not strongly associated with the pathogenesis of viral hepatitis and other hepatic disorders and did not differ significantly between the 2 groups.

### 3.3. Causal relationships between CAD and viral hepatitis in MR

After following the assumptions of independence, association and exclusivity when using SNPs as IVs, we screened 18 SNPs, 11 SNPs, and 19 SNPs from the GWAS datasets of phenotype MI, heart attack/MI and CHD as IVs for MR studies, respectively, all of which passed the *P*-value and linkage disequilibrium tests and met the significant criteria for genome-wide association. We confirmed through the PhenoScanner database that all IVs were not associated with confounders and did not have pleiotropic associations. Table S1, Supplemental Digital Content lists the details of each SNP.

**Figure 2.**
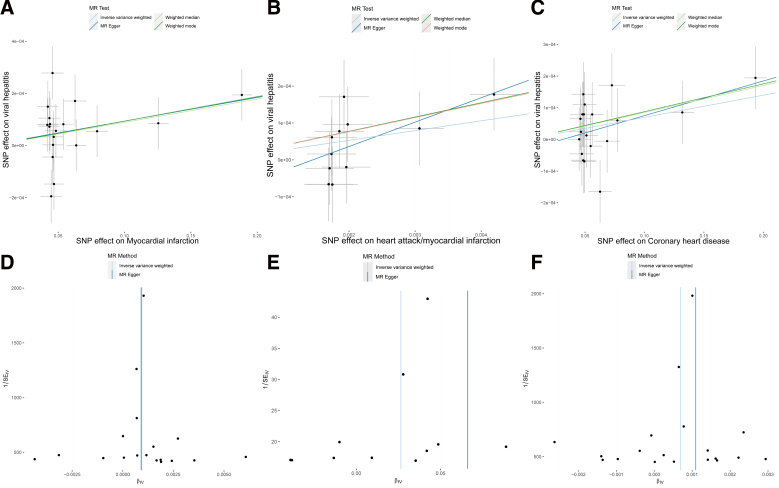
The scatter-plot (A–C) with the slope of each line representing the direction and strength of causality for each method and the funnel plot which assessing the heterogeneity between samples of myocardial infarction (D), heart attack/myocardial infarction (E), and coronary heart disease (F). Each point in the figure represents an instrumental variable, and the better the symmetry of the instrumental variable’s distribution, the lower the heterogeneity between the two samples. Note: SE_IV_: standard error of each instrumental variable; β_IV_: beta value of each instrumental variable. MR = Mendelian randomization, SNP = single nucleotide polymorphism.

In the forward MR analysis, in order to quantify the causal relationship between the 3 CAD-related phenotypes and viral hepatitis, we used 4 methods, including IVW, MR-Egger, weighted median and weighted mode, and IVW method was used as the main analysis method and judgement criteria. The results indicated a significant positive causal effect between the exposure factors MI (OR = 1.00094; 95% CI: 1.00026–1.00163, *P* = .007), heart attack/MI (OR = 1.027; 95% CI: 1.00065–1.054, *P* = .044), CHD (OR = 1.00070; 95% CI: 1.00010–1.0013, *P* = .022), and the viral hepatitis, also the results of the weighted median method were all significant for 3 CAD-related phenotypes (*P* < .05). It is noteworthy that in the forward MR analyses, the OR of all MR methods were in the same direction (OR > 1). Meanwhile, the results of reverse MR analysis suggested that there was no reverse causal association between the 3 CAD-related phenotypes and the viral hepatitis (*P* > .05; Table 4). By combining the 4 analyses, the results of the MR analysis further supported the potential causal relationship that the occurrence of CAD may contribute to the risk of developing viral hepatitis. In addition, we plotted both scatterplots (Fig. 2A–C) and forest plots (Fig. S1, Supplemental Digital Content) for each IV’s effect on viral hepatitis.

**Table 4:** Estimated causal effects of myocardial infarction on viral hepatitis by 4 MR methods.

Exposure	Outcome	Methods	SNPs	Beta	SE	*P*	OR (95% CI)	*P* of Reverse MR (IVW method)
Myocardial infarction (Sample size = 639,221)	Viral hepatitis (Sample size = 462,933)	Inverse variance weighted	18	0.000943224	0.000348	.006703955	1.000943669 (1.000261377–1.001626427)	.5430
		MR Egger	18	0.000906442	0.000702	.215096141	1.000906853 (0.999530231–1.002285371)	
		Weighted median	18	0.000890576	0.000441	.043303147	1.000890973 (1.000026784–1.001755909)	
		Weighted mode	18	0.000934269	0.000402	.032869615	1.000934705 (1.000145858– 1.001724175)	
Heart attack/Myocardial infarction (Sample size = 337,159)		Inverse variance weighted	11	0.026463799	0.013169	.044476967	1.026817075 (1.000652965–1.053665299)	.7244
		MR Egger	11	0.066074975	0.039700	.130402599	1.068306811 (0.988330686–1.154754637)	
		Weighted median	11	0.038696148	0.017592	.027828620	1.039454596 (1.004225503–1.075919556)	
		Weighted mode	11	0.038202271	0.020646	.093993867	1.038941359 (0.997738348–1.081845907)	
Coronary heart disease (Sample size = 184,305)		Inverse variance weighted	19	0.000697566	0.000304	.021646541	1.000697810 (1.000102221–1.001293753)	.2465
		MR Egger	19	0.001093254	0.000628	.099774619	1.001093853 (0.999862390–1.002326832)	
		Weighted median	19	0.000851988	0.000416	.040511167	1.000852351 (1.000036808–1.001668559)	
		Weighted mode	19	0.000877541	0.000440	.061543951	1.000877926 (1.000014900–1.001741697)	

95% CI = 95% confidence interval, Beta = beta value, IVW = inverse variance weighting, MR = Mendelian randomization, OR = odds ratio, *P* = *P* value, SE = standard error, SNP = single nucleotide polymorphism.

### 3.4. Sensitivity analysis and heterogeneity test

We examined the results of the IVW and MR-Egger methods for the 3 CAD-related phenotypes using Cochran *Q* test, with the level of significant heterogeneity setting at *P* < .05. The results of the test indicated that there was no heterogeneity between the IVs participating in the MR analyses for any of the 3 CAD-related phenotypes (*P* > .05; Table 5). Meanwhile, the *P*-value > .05 in both MR-PRESSO global test and MR-Egger intercept test suggested that there was no effect of horizontal pleiotropy in the whole analysis, MR-Egger intercept did not show any directional pleiotropy (Table 5). Furthermore, the funnel plot exhibited a symmetrical distribution of the causal impact of each SNP revolving around the IVW estimation, suggesting minimal susceptibility to horizontal pleiotropy altering the causal relationship (Fig. 2D–F), while the MR-PRESSO test did not reveal the presence of SNPs with abnormal outliers, and the leave-one-out analysis showed that none of the SNPs affected the overall estimation of the direction of the causal relationship, verifying the results of the present study (Fig. S2, Supplemental Digital Content).

**Table 5:** The results of sensitivity analysis and heterogeneity test.

Exposure	Outcome	Heterogeneity test				Pleiotropy test			
		Method	Q	Q_df	Q_pval	Method	Intercept	SE	*P*_val
Myocardial infarction (Sample size = 639,221)	Viral hepatitis (Sample size = 462,933)	IVW	19.495	17	0.301	MR Egger	0.00000311	0.000051	.952
		MR Egger	19.490	16	0.244	MR-PRESSO	N/A	N/A	.404
Heart attack/Myocardial infarction (Sample size = 337,159)		IVW	5.754	10	0.835	MR Egger	−0.0000960	0.0000907	.318
		MR Egger	4.635	9	0.865	MR-PRESSO	N/A	N/A	.856
Coronary heart disease (Sample size = 184,305)		IVW	12.152	18	0.839	MR Egger	−0.0000341	0.0000473	.481
		MR Egger	11.634	17	0.822	MR-PRESSO	N/A	N/A	.845

df = degree of freedom, IVW = inverse variance weighting, MR = Mendelian randomization, *P*_val = *P* value, SE = standard error.

## 4. Discussion

We integrated a cross-sectional study based on nationally representative NHANES 2017 to 2018 data and MR analyses based on 4 estimation methods (IVW, MR Egger, weighted median, and weighted mode) to explore the relationship between CAD and viral hepatitis. We firstly analyzed the NHANES 2017 to 2018 data to narrow down the scope of this study to the relationship between CAD and hepatic disorder including viral hepatitis, and the results of our cross-sectional study indicated a higher prevalence of CAD in the hepatic disorder group when compared to the control group. We also used a population-weighted regression analysis to confirm that CAD is a risk factor for hepatic disorder including viral hepatitis. However, due to the limitations of the questionnaire or interview method itself, it would be more difficult to determine the causal relationship between CAD and the viral hepatitis separately in cross-sectional study, therefore, we further used a bidirectional two-sample MR method to extract the causal relationship between CAD and viral hepatitis precisely, and the MR method further confirmed the positive causal effect of CAD on viral hepatitis, suggesting that CAD may be a risk factor for viral hepatitis.

This study’s primary strengths lie in its implementation of stringent measures to control bias and systematic analytical approaches, utilizing various models to evaluate the impacts of causality and produce reliable and consistent findings. We believe that the key to determining causality lies in the sequential relationship between the pathogenesis of these 2 disorders, which is based on the genetic central dogma, whereby human genotypes emerge before phenotypes, which gave us a completely new ways of thinking, the results of this study suggest that rs2954021. The specific allele information of rs2954021 includes that alleles: A/T (biallelic SNP), reference allele (Ref): T, alternative allele (Alt/effect/risk allele): A. rs2954021(A/T) is an IV that located near the *TRIB1* gene, may be a bridge that closely links CAD to viral hepatitis. In previous studies in different populations, rs2954021(A/T) has been shown to be significantly associated with nonalcoholic fatty liver disease at the histological level.^[51,52]^ Additionally, *TRIB1* also plays an important role in lipid metabolism in human hepatocytes,^[53]^ and a GWAS study showed that nonalcoholic fatty liver disease marked by the *TRIB1* locus (containing rs2954021[A/T]) is characterized by fat accumulation in the liver (hepatic steatosis).^[54]^ Hepatic steatosis is not only a risk factor necessary for the development and progression of hepatitis C-related diseases,^[55]^ but also closely associated with chronic hepatitis B.^[56]^ And steatosis has been shown to negatively impact hepatic antiviral therapy.^[57]^ Various evidence suggests that *TRIB1* rs2954021(A/T) is highly correlated with cytopathic effects induced by viral hepatitis at the morphological level, and due to the strong correlation between rs2954021(A/T) and CAD, CAD supports replacing rs2954021(A/T) as an exposure factor to increase the risk of viral hepatitis.

The findings of this study illustrate a causal relationship between the infection of viral hepatitis and CAD status, although the causality is not established in the reverse MR analysis (*P* > .05). However, we believe that the reverse MR analysis existed to ensure the requirement of uniqueness in MR studies for the direction of causality, which is not contradictory to the results of extant observational studies,^[58–61]^ instead, due to ethical requirements and varied objective conditions, it is a valuable complement to the existing studies in the absence of relevant prospective cohort studies. Moreover, as an infectious disease, viral hepatitis can’t contract without the involvement of hepatitis viruses, while CAD doesn’t directly cause viral hepatitis, which inspires us to focus on this question from an indirect perspective: could CAD provide the appropriate conditions for the infection of viral hepatitis? In this process, we considered that inflammation-associated molecules are an important pathway linking between CAD and viral hepatitis, and emerging evidence suggests that neutrophil extracellular traps (NETs) generated by activated neutrophils establishes a connection between inflammation and thrombosis.^[62]^ Different types of clinical studies have found a positive correlation between the level of NETs in blood and the degree of inflammation in MI patients.^[63,64]^ For viral hepatitis, Li et al found that NETs increased the hepatic fibronectin deposition while promoting inflammatory responses, and experiments demonstrated that NETs triggered by the FGL2-MCOLN3 autophagy axis could exacerbate liver injury in fulminant viral hepatitis.^[65]^ Thus, the inflammatory load may be mediated by NETs from the myocardium to the liver, promoting an inflammatory state in liver and increasing the probability of viral hepatitis infection. Protein molecules are equally important when discussing the effects of the heart on the liver. Syndecan-1 (SDC-1) protein has a tendency to regulate inflammation.^[66,67]^ Meanwhile, SDC-1 is a receptor for HCV, which is not only associated with HCV infection,^[68,69]^ but also highly expressed in HCV-infected livers,^[70]^ as SDC-1 molecules originating from hepatocyte cell membranes could promote HCV invasion, heralding long term chronic infection and multiple complications.^[71]^ In contrast, blocking SDC-1 expression could inhibit HCV invasion.^[68]^ Following MI in patients, excessive formation of circulating NETs as a potential contributor to endothelial functional and structural damage,^[63,72]^ lead to injury of the hepatocyte glycocalyx, resulting in elevated circulating SDC-1 levels.^[73,74]^ Since the specific microenvironment containing SDC-1 near the hepatocyte membrane is one of the reasons for the hepatotropism of HCV,^[68]^ we speculate that MI may indirectly involve the liver by enhancing the invasiveness of hepatitis virus to hepatocytes through elevating the levels of NETs and SDC-1 in the bloodstream. The concomitant presence of NETs and SDC-1 in pathological process is not an isolated case, as one of the characteristics of MI, endothelial injury is also a key trigger of sepsis-induced coagulopathy (SIC).^[75]^ A previous clinical study showed that patients with sepsis combined with DIC had significantly higher NETs markers and SDC-1 in their blood compared to controls without DIC. And the formation of NETs was significantly associated with syndecan-1.^[76]^ However, we still need more evidence to explain the mechanism by which CAD promotes the internalization of hepatitis viruses. Additionally, within the healthcare environment surrounding CAD patients, they may undergo more medical interventions (hospitalization, invasive procedures, and blood transfusions), which could increase the risk of contracting blood-borne infections such as viral hepatitis. For instance, HBV can be transmitted through blood exposure during medical procedures like dialysis and surgery, accidental contact such as needle stick injuries,^[77]^ and previous reports have also noted the possibility of HCV infection through open-heart surgery.^[78]^

Comorbidities cannot be ignored when discussing the association of CAD with viral hepatitis. Diabetes as a comorbidity also occurs in patients with viral hepatitis, a retrospective analysis which conducted on 1117 patients diagnosed with chronic viral hepatitis revealed that 21% of patients infected with HCV had diabetes, while 12% of patients infected with HBV had the same comorbidity (*P* = .0004; 95% CI: 1.3–2.4). In another case-control trial in the same report, the prevalence of HCV infection was significantly higher in diabetic patients than in controls (4.2% vs 1.6%; *P* = .02).^[79]^ Another cohort study in the Asia-Pacific region included 7149 patients with chronic hepatitis C, of whom 722 (10.1%) were co-infected with HBV. Among them, 1589 (22.2%) patients had DM, and the prevalence of DM was similar in the mono-infected and dual-infected cohorts (22.3% vs 21.3%).^[80]^ The strong association between diabetes and viral hepatitis is consistent with what we observed from the cross-sectional study in NHANES. These data suggest that it is not only the 2 diseases themselves that need to be guarded against in patients with poor prognosis in the CAD population and those at high risk of viral hepatitis, but also other systemic symptoms that may act as comorbidities and inversely contribute to inflammatory damage, and we need more effective means of surveillance and prevention to counter the threat to patients’ quality of life and survival time.

## 5. Conclusions

Through a cross-sectional study, we first established a significant association between broadly defined hepatic disorder (including viral hepatitis) and CAD/CAD-related conditions. Subsequently, using MR analysis, we uncovered potential evidence of a causal relationship between CAD and the onset of viral hepatitis. The primary significance of this study lies in its support for using CAD as a marker to identify high-risk populations for viral hepatitis infection. It facilitates the development of public health policies targeting viral hepatitis towards early prevention.

## 6. Limitation

This study has several limitations. Firstly, the NHANES study is only representative of the US population, and the GWAS data used in MR study is mainly from the European population, among them, all samples in the ukb-a-63 and ukb-b-7901 datasets are from participants of British ancestry. The proportion of participants explicitly identified as European ancestry in the ebi-a-GCST011365 dataset is 61.92%, and the proportion of European ancestry participants in the ieu-a-7 dataset is 77%. Within the MR framework, this implies that if we detect a causal effect in a sample where Europeans constitute overwhelming majority of participants, the IV may lose its explanatory power for the exposure factor in smaller non-Europeansubgroups. Consequently, the causal estimates for these non-European subgroups become unreliable. Interpreting such findings as universal principles applicable to all humanity could be misleading.^[81,82]^ Secondly, in the NHANES section, diagnostic information on viral hepatitis and CAD was obtained by means of personal interviews and questionnaires, although we controlled many of the known confounders in our process, the results of the NHANES section may be affected by recall bias due to the nature of a cross-sectional study, furthermore, the data used for cross-sectional study and the GWAS summary data did not separately provide the information on viral hepatitis subtypes such as A, B, C and the corresponding clinical phenotypes such as acute or chronic, mild or severe course, especially the lack of information regarding whether participants had received antiviral or cardiovascular treatments. The last but not the least, although CAD may offer appropriate conditions for the infection or onset of viral hepatitis, it must be recognized that the foremost causative factor of viral hepatitis is the infection with hepatitis virus, various factors such as lifestyle, occupation, and underlying diseases may affect the severity of viral hepatitis, we cannot ignore the residual confounding effects caused by shared risk factors, particularly differences in socioeconomic status. In this study, dynastic effects rooted in socioeconomic status may amplify or distort the causal effects estimated by MR analysis.^[83]^ For instance, if parental genetic variation influences their socioeconomic status, which in turn affects offspring health outcomes by providing better medical resources, the observed association between genetic variation and offspring health outcomes may not be purely causal. Instead, it could be confounded by intergenerational cumulative effects. Although our findings suggest a potential causal relationship between CAD and the risk of viral hepatitis, since the results of this study were based on computerized statistical analyses, more basic medical research is needed to elucidate the specific mechanisms by which CAD may influence the emergence of viral hepatitis. Moreover, In the cross-sectional study, the number of viral hepatitis cases available for sensitivity analysis is insufficient, this is because the NHANES 2017 to 2018 does not make specific viral hepatitis subtype data publicly accessible, and we were unable to locate specific hepatitis patient ID numbers within the hepatic disorder entries, therefore, we cannot definitively determine the number of people diagnosed with viral hepatitis.

We used the mRnd network computation tool (https://shiny.cnsgenomics.com/mRnd/) to estimate the statistical power of the MR analysis in this study. To avoid the true OR of the outcome variable per standard deviation of the exposure variable being too small, which would lead to bias in the calculation of statistical power, we set the OR of all 3 outcome variables to 1.1. If we select a smaller ratio (e.g., 1.001), it would imply an excessively weak effect, which requiring an impractically large sample size to achieve sufficient statistical power. This may be inconsistent with existing observational evidence and fall outside the intended scope of our study.^[84]^ After calculation, The MR statistical power of ebi-a-GCST011365 is 0.91, the MR statistical power of ukb-a-63 is 0.23, and the MR statistical power of ieu-a-7 is 0.79. It is worth noting that all associations identified through MR analyses in this study were of modest magnitude. Although the effects were weak, at the large population level, even when the OR is so close to 1, significant cumulative benefits can still be achieved if the prevalence of the exposure factor such as CAD is high and the factor is amenable to intervention. Nonetheless, the power of MR studies primarily relies on the robustness of the relationship between IV and risk factors,^[85]^ the F-statistics of all the incorporated SNPs exceeded 10, suggesting that our causal inference retains a certain level of credibility.

## Acknowledgments

The authors would like to thank all participants and researchers for contributing and sharing GWAS summary data. Figure 1 was drawn by Figdraw. NHANES data processing and analysis were performed using Zstats 1.0.

## Author contribution statement

**Conceptualization:** Xiuli Men, Zihan Zhou.

**Data curation:** Zihan Zhou.

**Formal analysis:** Zihan Zhou.

**Funding acquisition:** Xiuli Men.

**Investigation:** Zihan Zhou.

**Methodology:** Xiuli Men, Zihan Zhou.

**Software:** Zihan Zhou.

**Supervision:** Xiuli Men, Jing Wu, Lan Li, Aibin Cheng.

**Validation:** Zhuo Bai.

**Visualization:** Chang Zheng, Zhuo Bai.

**Writing – original draft:** Zihan Zhou, Zhuo Bai, Chang Zheng.

**Writing – review & editing:** Zihan Zhou, Jing Wu, Lan Li, Aibin Cheng, Xiuli Men.



**Figure:**
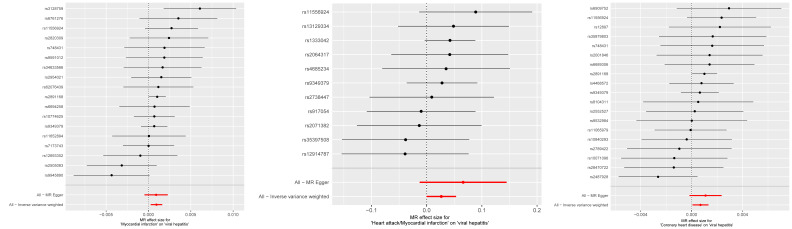


**Figure:**